# Ascites promotes cell migration through the repression of miR-125b in ovarian cancer

**DOI:** 10.18632/oncotarget.16846

**Published:** 2017-04-05

**Authors:** Lan Yang, Xiaoli Zhang, Yiming Ma, Xinhua Zhao, Bin Li, Hongying Wang

**Affiliations:** ^1^ State Key Laboratory of Molecular Oncology, National Cancer Center/Cancer Hospital, Chinese Academy of Medical Sciences and Peking Union Medical College, Beijing, PR China; ^2^ Department of Gynecological Oncology, National Cancer Center/Cancer Hospital, Chinese Academy of Medical Sciences and Peking Union Medical College, Beijing, PR China

**Keywords:** ovarian cancer, ascites, cell migration, TGF-β, miR-125b

## Abstract

Interactions between ovarian cancer cells and the surrounding tumor microenvironment are not well characterized. Here, we investigated the molecular mechanisms by which malignant ascites promote the metastasis of ovarian cancer. It was found that ovarian cancer ascites promoted ovarian cancer cell migration which was attenuated by either heat inactivation or antibody blockade of TGF-β. High level (at ng/ml level) of TGF-β was detected in the ascites. In addition, ascites repressed the expression of miRNA-125b in a TGF-β-dependent manner. Mimic of miR-125b blocked ascites-induced cell migration. Furthermore, Gab2 (a target gene of miR-125b) was elevated by ascites in a TGF-β-dependent manner. And forced expression of Gab2 reversed the inhibition of migration induced by miR-125b mimic. Most importantly, the expression of miR-125b and Gab2 mRNA was negatively correlated in ovarian cancer specimens. Taken together, our finding suggested that TGF-β in ascites promoted cancer cell migration through repression of miR-125b in ovarian cancer. This might provide a novel therapeutic target for ovarian cancer in the future.

## INTRODUCTION

Ovarian cancer is the third common malignancy in female reproductive system. Despite enormous progress in cancer biology, ovarian cancer is one of the leading causes of cancer death among women worldwide due to the advanced stage of disease at diagnosis. Ovarian cancer is a highly metastatic cancer characterized by widespread intraperitoneal dissemination. It is well accepted that cancer metastasis is the major causes of the failure of cancer treatment. The clarification of molecular mechanisms regulating this process is vital for the development of an efficient therapy for ovarian cancer especially considering the early-stage detection is still a conundrum.

Cancer metastasis is a multi-step process and regulated by a complex interaction between cancer cells and their microenvironment. Due to the lack of an anatomical barrier, ovarian cancer can spread directly throughout the peritoneal cavity mainly by intra-abdominal and lymphatic dissemination, and metastasize to peritoneum and omentum [1]. Because of the same reason, ovarian cancer is prone to be regulated by the signaling from peritoneal cavity, such as ascites. It is well recognized that the presence of ascites is a poor prognostic indicator for survival in ovarian cancer. Five-year survival rate dramatically dropped from 45% to 5% when ascites appeared among women with stage III or IV epithelial ovarian carcinoma [2]. Even in women with stage I disease, ascites has been found to be a poor prognostic factor [3]. Although ascites is associated with cancer progression [4], the mechanisms by which ascites regulates the metastasis in ovarian cancer cells is not fully understood.

MicroRNAs (miRNAs) are approximately ~22 nucleotide (nt) RNA molecules that modulate gene expression by translational repression and/or degradation of mRNA, and are actively involved in the progress of metastasis and invasion in cancer [5]. Deregulation of miR-125b has been associated with metastasis and the poor prognosis [6, 7]. through targeting specific genes such as MTA1 (metastasis-associated gene 1) [8], Lin28B (Lin-28 homolog B) [6] and Gab2 (Grb2-associated binding protein 2) [9] in various cancers. In current study, we investigated whether ascites regulate ovarian cancer cell migration through miR-125b and which component in ascites plays this effect on ovarian cancer cells.

## RESULTS

### TGF-β involved in ascites-induced ovarian cancer cell migration

**Figure 1:**
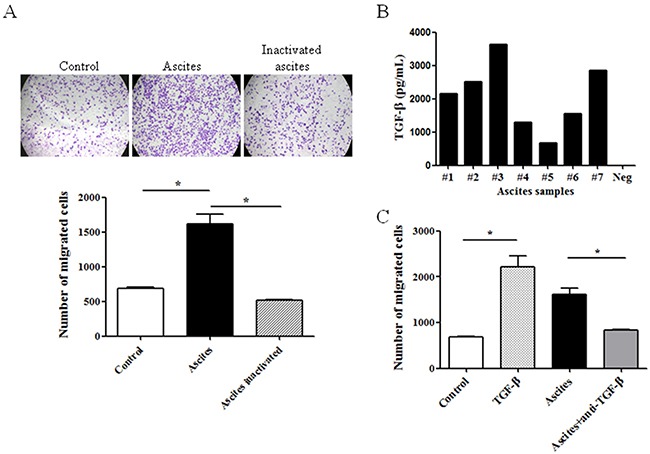
Ascites promoted ovarian cancer cell migration in a TGF-β-dependent manner **(A)** Cells were seeded on transwell and migrated cells were stained and quantified after treatment with the ascites or inactivated ascites (heated at 100°C for 10 min) for 10 hours. **(B)** The level of activated TGF-β in seven different ascites specimens (#1-#7) was measured with ELISA kit. Neg, negative control. **(C)** Same as **(A)**, after 10-hour treatment with TGF-β, ascites or ascites conbined with anti-TGF-β antibody, migrated cells were quantified. The data showed the means ± SD from three independent experiments. *p < 0.05 vs corresponding control.

In the study, human ovary carcinoma-derived epithelial cell lines TOV-112D and SKOV-3 were used to test the effect of ascites on cell migration. It was shown by transwell assay that treatment with ascites for 10 hours significantly promoted cell migration in TOV-112D cell line (Figure 1A). However, heat treatment of ascites almost completely abrogated ascites-induced cell migration (Figure 1A). Since TGF-β is a potent stimulator of cell migration in different cancers [10, 11], we hypothesized that it might be TGF-β in ascites to stimulate cell migration. Firstly, TGF-β at ng/ml level was detected in all ascites sample we tested (Figure 1B). Most importantly, pretreatment with anti-TGF-β antibody dramatically abolished stimulatory effect of ascites on cell migration (Figure 1C). The same results were observed in SKOV-3 cell (data not show). The results indicated that TGF-β might contribute ascites-induced cell migration in ovarian cancer.

### Repression of miR-125b mediated ascites induced cell migration

**Figure 2:**
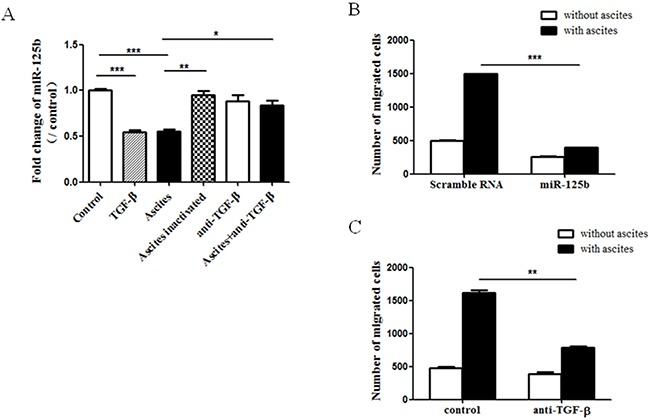
MiR-125b mediated ascites- and TGF-beta-induced ovarian cancer cell migration **(A)** After the treatment with TGF-β, ascites, inactivated ascites and ascites conbined with anti-TGF-beta antibody for 6 hours, cells were collected and tested for miR-125b expression by real-time PCR. **(B)** Thirty-six hours after transfection with miR-125b or scramble RNA, the cells were seeded into transwell and incubated with or without ascites for 10 hours. Migrated cells were quantified. **(C)** Cells on transwell were treated with ascites for 10 hours combined with or without anti-TGF-β antibody. Migrated cells were quantified. The data showed the means ± SD from three independent experiments. *p < 0.05, **p < 0.01, ***p < 0.001 vs corresponding control.

MiRNAs are emerging as important regulators of gene expression by inhibiting their translation [12, 13]. In previous study, we found that down-regulation of miR-125b promoted colorectal cancer cell migration [9]. To test whether the miR-125b involved in ascites-related migration, we measured the level of miR-125b. It was found that incubation with ascites, same as TGF-β (5μg/mL), repressed the level of miR-125b in ovarian caner cell (Figure 2A). Inactivated ascites with heat did not show any effect on miR-125b (Figure 2A). Forced expression of miR-125b blocked ascites-induced cell migration compared with scramble RNA control group (Figure 2B). Pretreatment with anti-TGFβ antibody not only restored repression of miR-125b (Figure 2A) but also abolished cell migration induced by ascites in TOV-112D cells (Figure 2B). Taken together, the findings indicated that repression of miR-125b mediated ascites-induced cell migration.

### Gab2 mediated ascites induced cell migration

**Figure 3:**
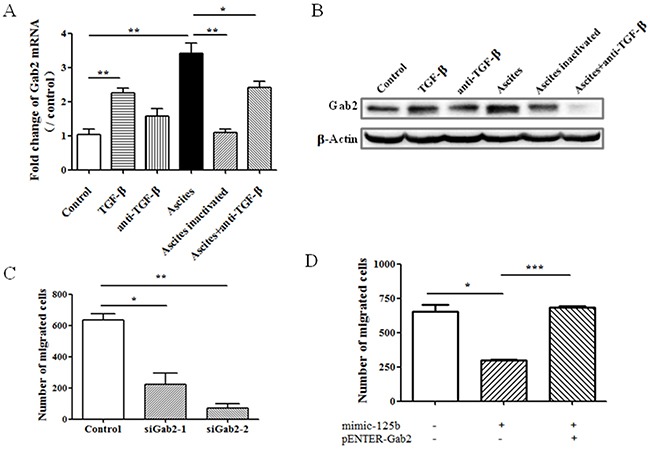
Gab2 mediates miR-125b-related cell migration in ovarian cancer cell After treatment for 10 hours, the expression of Gab2 **(A)** mRNA and **(B)** protein in cells was measured with real time PCR and western blot respectively. Cells were transfected with **(C)** siRNA against Gab2, or **(D)** miR-125b (mimic-125b) with or without Gab2-expressing plasmid (pENTER-Gab2). Thirty-six hours after transfection, the cells were seeded into transwell and treated with or without ascites for another 10 hours. Migrated cells were quantified. The data showed the means ± SD from three independent experiments. *p < 0.05, **p < 0.01, ***p < 0.001 vs corresponding control.

In previous study, we have identified Gab2 as miR-125b target gene that functions to mediate cell migration in colorectal cancer [9]. Therefore we tested whether Gab2 mediates ascites-induced cell migration in ovarian cancer cells. Firstly, ascites significantly enhanced the expression of Gab2 at mRNA level which was blocked by inactivation with heat and anti-TGF-β antibody (Figure 3A). The change of Gab2 protein was consistent with that of mRNA (Figure 3B). Specific siRNAs against Gab2 were applied to reduce the expression of Gab2 at both mRNA and protein levels (data not shown). Knockdown of Gab2 significantly blocked ascites-induced cell migration (Figure 3C). In addition, overexpression of Gab2 completely reversed the repression of cell migration induced by miR-125b (Figure 3D). Therefore, these findings indicated that miR-125b targeted Gab2 and mediated cell migration induced by ascites.

### Correlation of miR-125b and Gab2 in human ovarian cancer

**Figure 4:**
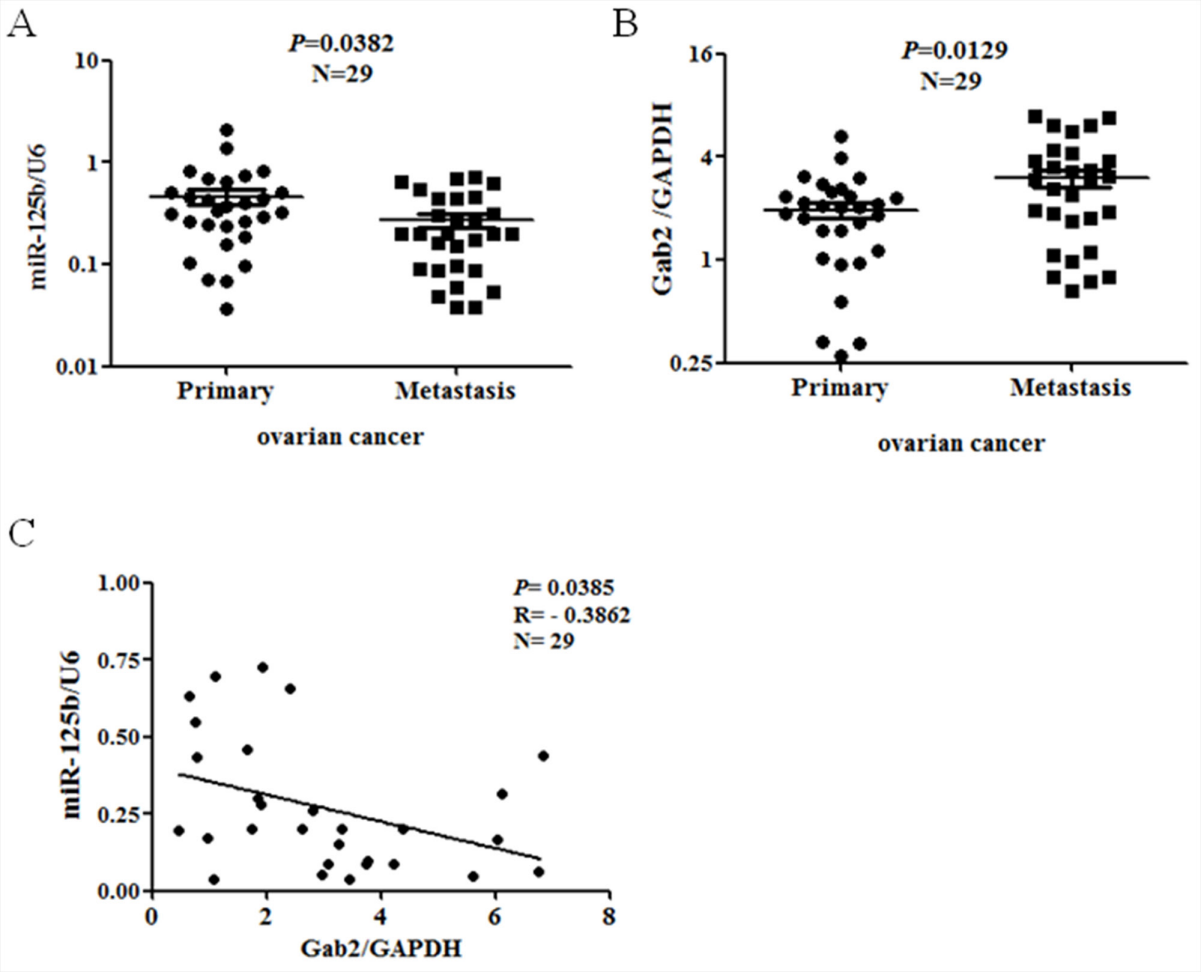
Correlation of miR-125b and Gab2 in ovarian cancer specimens The expression of miR-125b and Gab2 in paired primary and metastatic human ovarian cancer specimens was tested with real time PCR. The level of **(A)** miR-125b and **(B)** Gab2 were normalized with U6 or GAPDH respectively. **(C)** Pearson's correlation analysis was conducted for Gab2/miR-125b. Black line represents linear regression line.

To confirm the findings *in vivo*, the expression of miR-125b and Gab2 in 29 paired human ovary cancer specimens was measured by real time PCR. As shown in (Figure 4A and 4B), miR-125b was downregulated while Gab2 was upregulated in metastasis group compared with primary group. Importantly, the level of miR-125b was negatively correlated with the level of Gab2 in metastatic samples (Figure 4C). It strongly indicated that ascites might stimulate metastasis through miR-125b/Gab2 in human ovarian cancer.

## DISCUSSION

Malignant ovarian ascites is rich in proteolytic enzymes, including MMP-2, MMP-9, and uPA [14, 15], biologically active lipid factors, such as lysophosphatidic acid (LPA) [16], a variety of cytokines, growth factors, and also hormones [17]. The presence of proteolytic enzymes in ascites and the correlation between ascites and poor prognosis suggest that this complex fluid may play an active role, rather than a passive role, in ovarian metastasis. In deed, malignant ascites is known to protect human ovarian cancer cells from TRAIL-induced apoptosis through αvβ5 integrin-mediated focal adhesion kinase/Akt activation and ERK1/2-Elk-1 signaling [17, 18]. In addition, LPA in ascites has been shown to promote migration [19] and invasion of epithelial ovarian cancer cells [20, 21]. However, in current study, LPA does not seem to contribute to ascites-induced cell migration because LPA is relatively heat resistant [22]. We found that high TGF-β expression at ng/ml level was detected in all malignant ascites samples tested, which is consistent with previous report [23]. All samples were tested after acidification and the detected values concerned total TGF-β including active and latent form. Of note, no TGF-β in active form was detectable in any of the samples without acidification. Thus it is reasonable that the effect of TGF-β in ascites lies on the ability of cancer cell to locally activate latent TGF-β.

In the last decade, miRNAs have emerged as critical regulators in various cancers, including ovarian cancer [24]. The role of miR-125b in tumorigenesis is controversial since miR-125b can act as oncogene or tumor suppressor, depending on tumor types. In ovarian cancer, miR-125b has been shown to be downregulated and acts as a tumor suppressor [24, 25]. Consistently miR-125b suppressed the oncogenicity of ovarian cancer cells in nude mice. Furthermore, ectopic expression of miR-125b in ovarian cancer cells induced cell cycle arrest and led to reduction in proliferation and clonal formation through the repression of a proto-oncogene, BCL3 [25]. In addition, PPARγ can induce growth suppression of ovarian cancer by transcriptional upregulation of miR-125b and thereby inhibits proto-oncogene BCL3 [26]. Furthermore, miR-125b inhibited invasion and migration of ovarian cancer cells through posttranscriptional down-regulation of EIF4EBP1 expression [27]. In present study, we found that the expression of miR-125b was downregulated in metastasis sites compared with primary tumor. This observation is consistent with previous finding that the level of miR-125b in tumor tissue went down with the progression of ovarian cancer [28]. However, serum level of miRNA-125b was significantly higher in epithelial ovarian cancer patients compared to health or benign controls, and especially was higher in early stages I and II. Notably, the higher level of miR-125b in serum was significantly correlated with better progression-free survival rate [28, 29].

Most interestingly, TGF-β is also involved in the formation of ascites. The accumulation of ascites is caused by an imbalance between fluid extravasation from the blood vessels and re-absorption by lymphatic vessels. Vascular Endothelial Growth Factor (VEGF) is crucial for the production of malignant ascites [30]. TGF-β blockade not only inhibited ascites production via inhibition of VEGF, but also improved ascites drainage in an orthotopic human ovarian carcinoma model [31]. Although TGF-β blockade decreased lymphangiogenesis, it maintained the normal lymphatic vessel morphology and valve structure resulting improved the drainage function of diaphragm lymphatic vessels [31]. In addition, TGF-β blockade has been shown to recruit pericyte and induce blood vessel “normalization” [32]. Thus, it is reasonable to consider the use of TGF-β blockade to inhibit cancer metastasis and also control ascites.

Actually, the interaction between TGF-β and miR-125 has been showed in other cancers. Suppression of miR-125b mediated TGF-β-induced EMT in hepatocellular carcinoma (HCC) [33]. Moreover, miR-125b attenuated EMT-associated chemoresistance, migration, and stemness of HCC both *in vitro* and *in vivo* [33]. These findings suggest a potential therapeutic treatment of miR-125b for liver cancer. On the other hand, miR-125b, miR-99a/100 and let-7 are encoded in two tricistrons on human chromosomes 11 and 21 [34]. They are highly expressed in hematopoietic stem cells (HSCs). Notably, miR-125b, miR-99a/100 and let-7 can switch the balance between TGF-β and Wnt signaling in order to regulate hematopoietic stem and progenitor cell homeostasis [34]. Thus, it is possible that miR-125b regulated by TGF-β in ascites might play an important role in the malignant behavior of ovarian cancer.

Taken together, our finding suggested that TGF-β in ascites promoted cell migration through repression of miR-125b in ovarian cancer. It not only reveals a novel molecular mechanism underlying promotion of metastasis by malignant ascites but also provides a potential target for development of new therapeutic approaches against ovarian cancer.

## MATERIALS AND METHODS

### Cell culture and treatment

Human ovarian epithelial cell lines TOV-112D and SKOV-3 were purchased from ATCC (Manassas, VA, USA). The cells were grown in Dulbecco's modified Eagle's medium/F12 (Hyclone) supplemented with 10% fetal bovine serum (Gibco).

MiR-125b mimic, inhibitor and negative control Oligos were obtained from GeneCopoeia (GuangZhou Ribobio Co. Ltd., China). SiRNA targeting Gab2(5’-GAG ACA GCG AAG AGA ACU ATT-3’) was purchased from Genepharma (Suzhou, China).

### RNA isolation and real-time PCR

Total RNA was isolated from cell lines, patient samples with Trizol reagent (Invitrogen). After treatment with DNase I, RNA was reverse transcribed into cDNA with Thermo scientific maxima first strand cDNA synthesis kit for mRNA detection, or with Takara™ microRNA transcription kit for microRNA detection. Real time PCR was carried out on Bio-Rad S1000 PCR instrument and each sample was analyzed in triplicate. PCR data were normalized to GAPDH or U6 snRNA expression for mRNA and miRNA respectively.

Primers for miR-125b and U6 were obtained from GeneCopoeia (GuangZhou Ribobio Co.Ltd, China), The antisense primer for miRNAs was bought from Takara (Dalian, China). Gab2 primers as follows: sense, 5’-CGC TGC TAGAC AAC AGC CGA CTT CAC C-3’ and antisense, 5’-GCC CAC AAT CAT TTT CCC T -3’.

### Western blot

Proteins were extracted from TOV-112D cells with RIPA buffer (0.01%EDTA, 0.1% Triton X-100 and 10% proteinase inhibitor cocktail). Protein concentrations were quantified using a protein assay kit (Bio-Rad). 100 μg of lysates were separated on 10% SDS-PAGE gel and transferred to polyvinylidene difluoride membranes. The membranes were probed overnight at 4°C with primary antibody against human Gab2 (Cell Signaling Technology, 1:1000), β-Actin (Sigma, 1:10000), followed by incubation with peroxidase-conjugated secondary antibody (Cell Signaling Technology) for 1.5 hours. The signal was visualized with ECL (Millipore).

### Migration assay

After starving in serum-free media for 12 hours, 5 × 10^4^ cells were seeded on the top chamber of transwell (6.5-µm pore; Corning, MA, USA) and incubated with ascites or TGF-β (5 μg/mL) and TGF-β antibody (final concentration 2 μg/mL) (Santa Cruz Biotechnology), while 600 µL media containing 10% FBS was added to the bottom chamber. Ten hours later, the cells remaining in the upper chamber were removed with cotton swabs and the cells on the undersurface of transwell were fixed and stained with crystal violet solution. The number of migratory cells was calculated by counting five different fields under a phase-contrast microscope of each transwell filter.

### Transduction with interferential miR-125b chemical and Gab2 plasmid

Full-length of Gab2 sequence and scrambled pENTER vector were purchased from Vigene (Rockville, MD, USA) and used as previously reported [9]. The cells in 6-well plates were transfected with miR-125b mimic (100 nM) combined with pENTER-Gab2. The transfection agent Lipofectamine 2000 (Invitrogen) was incubated with DNA or RNA in serum free media for 20 minutes before incubating 6 hours at 37°C, the supernatant was replaced with 2mL complete medium. Assessment of the effects of gene expression on the migration was performed 48 to 72 hours after transduction.

### Clinic tissue specimen

Ascites samples from 5 patients and all the ovarian cancer primary and metastasis sites tissue were obtained from Cancer hospital, Chinese Academy of Medical Sciences, Beijing, China. No patients received chemotherapy or radiotherapy before resection. Ascites samples without anticoagulants were centrifuged at 4,000 rpm for 20 minutes to separate fluid and cellular component, and the fluid was store at -70°C until usage. Ascites treated at 100°C for 10 min as a inactivated ascites group.

After obtaining informed consent and performing surgery at Cancer Hospital, Chinese Academy of Medical Sciences (Beijing, China), samples of primary ovarian cancer were taken and submerged in RNA later™ (Ambion) for 24h and then stored at -70°C until use. The use of human tissues was approved by the Institutional Review Board of the Chinese Academy of Medical Sciences Cancer Institute.

### Measurement of activated TGF-β

Level of TGF-β in ascites was measured with a Human TGF-β 1 Quantikine ELISA Kit (R&D systems). All samples were acidified with 1N HCl for 10 minutes and then active form of TGF-β was tested according to manufacture's recommendation.

### Statistical analysis

Statistical analyses were performed on data collected from at least three independent experiments. Data were presented as means±SD and analysis using GraphPad Prism 5 software. Comparison of >2 groups was made using ANVOA with Tukey test. Comparison of 2 groups was made using Student's *t* test for unpaired data. The differences were considered statistically significant when *p* value less than 0.05.

## References

[1] Vergara D, Merlot B, Lucot JP, Collinet P, Vinatier D, Fournier I, Salzet M (2010). Epithelial-mesenchymal transition in ovarian cancer. Cancer Lett.

[2] Puls LE, Duniho T, Hunter JE, Kryscio R, Blackhurst D, Gallion H (1996). The prognostic implication of ascites in advanced-stage ovarian cancer. Gynecol Oncol.

[3] Dembo AJ, Davy M, Stenwig AE, Berle EJ, Bush RS, Kjorstad K (1990). Prognostic factors in patients with stage I epithelial ovarian cancer. Obstet Gynecol.

[4] Huang H, Li YJ, Lan CY, Huang QD, Feng YL, Huang YW, Liu JH (2013). Clinical significance of ascites in epithelial ovarian cancer. Neoplasma.

[5] Ha M, Kim VN (2014). Regulation of microRNA biogenesis. Nat Rev Mol Cell Biol.

[6] Liang L, Wong CM, Ying Q, Fan DN, Huang S, Ding J, Yao J, Yan M, Li J, Yao M, Ng IO, He X (2010). MicroRNA-125b suppressesed human liver cancer cell proliferation and metastasis by directly targeting oncogene LIN28B2. Hepatology.

[7] Zhang Y, Yan LX, Wu QN, Du ZM, Chen J, Liao DZ, Huang MY, Hou JH, Wu QL, Zeng MS, Huang WL, Zeng YX, Shao JY (2011). miR-125b is methylated and functions as a tumor suppressor by regulating the ETS1 proto-oncogene in human invasive breast cancer. Cancer Res.

[8] Li Y, Chao Y, Fang Y, Wang J, Wang M, Zhang H, Ying M, Zhu X, Wang H (2013). MTA1 promotes the invasion and migration of non-small cell lung cancer cells by downregulating miR-125b. J Exp Clin Cancer Res.

[9] Yang L, Ma Y, Han W, Li W, Cui L, Zhao X, Tian Y, Zhou Z, Wang W, Wang H (2015). Proteinase-activated receptor 2 promotes cancer cell migration through RNA methylation-mediated repression of miR-125b. J Biol Chem.

[10] Qiu X, Cheng JC, Zhao J, Chang HM, Leung PC (2015). Transforming growth factor-β stimulates human ovarian cancer cell migration by up-regulating connexin43 expression via Smad2/3 signaling. Cell Signal.

[11] Xiang G, Yi Y, Weiwei H, Weiming W (2015). TGIF1 promoted the growth and migration of cancer cells in nonsmall cell lung cancer. Tumour Biol.

[12] Lin S, Gregory RI (2015). MicroRNA biogenesis pathways in cancer. Nat Rev Cancer.

[13] Ma Y, Li W, Wang H (2013). Roles of miRNA in the initiation and development of colorectal carcinoma. Curr Pharm Des.

[14] Dolo V, D’Ascenzo S, Violini S, Pompucci L, Festuccia C, Ginestra A, Vittorelli ML, Canevari S, Pavan A (1999). Matrix-degrading proteinases are shed in membrane vesicles by ovarian cancer cells *in vivo* and *in vitro*. Clin Exp Metastasis.

[15] Young TN, Rodriguez GC, Rinehart AR, Bast RC, Pizzo SV, Stack MS (1996). Characterization of gelatinases linked to extracellular matrix invasion in ovarian adenocarcinoma: purification of matrix metalloproteinase 2. Gynecol Oncol.

[16] Xu Y, Gaudette DC, Boynton JD, Frankel A, Fang XJ, Sharma A, Hurteau J, Casey G, Goodbody A, Mellors A, Holub BJ, Mills GB (1995). Characterization of an ovarian cancer activating factor in ascites from ovarian cancer patients. Clin Cancer Res.

[17] Lane D, Goncharenko-Khaider N, Rancourt C, Piché A (2010). Ovarian cancer ascites protects from TRAIL-induced cell death through v 5 integrin-mediated focal adhesion kinase and Akt activation. Oncogene.

[18] Goncharenko-Khaider N, Matte I, Lane D, Rancourt C, Piché A (2012). Ovarian cancer ascites increase Mcl-1 expression in tumor cells through ERK1/2-Elk-1 signaling to attenuate TRAIL-induced apoptosis. Mol Cancer.

[19] Ren J, Xiao YJ, Singh LS, Zhao X, Zhao Z, Feng L, Rose TM, Prestwich GD, Xu Y (2006). Lysophosphatidic acid is constitutively produced by human peritoneal mesothelial cells and enhances adhesion, migration, and invasion of ovarian cancer cells. Cancer Res.

[20] Graves LE, Ariztia EV, Navari JR, Matzel HJ, Stack MS, Fishman DA (2004). Proinvasive properties of ovarian cancer ascites-derived membrane vesicles. Cancer Res.

[21] Puiffe ML, Le Page C, Filali-Mouhim A, Zietarska M, Ouellet V, Tonin PN, Chevrette M, Provencher DM, Mes-Masson AM (2007). Characterization of ovarian cancer ascites on cell invasion, proliferation, spheroid formation, and gene expression in an *in vitro* model of epithelial ovarian cancer. Neoplasia.

[22] Mills GB, Moolenaar WH (2003). The emerging role of lysophosphatidic acid in cancer. Nat Rev Cancer.

[23] Yigit R, Figdor CG, Zusterzeel PL, Pots JM, Torensma R, Massuger LF (2011). Cytokine analysis as a tool to understand tumour-host interaction in ovarian cancer. Eur J Cancer.

[24] Nam EJ, Yoon H, Kim SW, Kim H, Kim YT, Kim JH, Kim JW, Kim S (2008). MicroRNA expression profiles in serous ovarian carcinoma. Clin Cancer Res.

[25] Guan Y, Yao H, Zheng Z, Qiu G, Sun K (2011). MiR-125b targets BCL3 and suppresses ovarian cancer proliferation. Int J Cancer.

[26] Luo S, Wang J, Ma Y, Yao Z, Pan H (2015). inhibits ovarian cancer cells proliferation through upregulation of miR-125b. Biochem Biophys Res Commun.

[27] Lee M, Kim EJ, Jeon MJ (2016). MicroRNAs 125a and 125b inhibit ovarian cancer cells through post-transcriptional inactivation of EIF4EBP1. Oncotarget.

[28] Zuberi M, Khan I, Gandhi G, Ray PC, Saxena A (2016). Utility of serum miR-125b as a diagnostic and prognostic indicator and its alliance with a panel of tumor suppressor genes in epithelial. PLoS One.

[29] Zhu T, Gao W, Chen X, Zhang Y, Wu M, Zhang P, Wang S (2016). A pilot study of circulating microRNA-125b as a diagnostic and prognostic biomarker for epithelial ovarian cancer. Int J Gynecol Cancer.

[30] Nagy JA, Masse EM, Herzberg KT, Meyers MS, Yeo KT, Yeo TK, Sioussat TM, Dvorak HF (1995). Pathogenesis of ascites tumor growth: vascular permeability factor, vascular hyperpermeability, and ascites fluid accumulation. Cancer Res.

[31] Liao S, Liu J, Lin P, Shi T, Jain RK, Xu L (2011). TGF-beta blockade controls ascites by preventing abnormalization of lymphatic vessels in orthotopic human ovarian carcinoma models. Clin Cancer Res.

[32] Salnikov AV, Roswall P, Sundberg C, Gardner H, Heldin NE, Rubin K (2005). Inhibition of TGF-beta modulates macrophages and vessel maturation in parallel to a lowering of interstitial fluid pressure in experimental carcinoma. Lab Invest.

[33] Zhou JN, Zeng Q, Wang HY, Zhang B, Li ST, Nan X, Cao N, Fu CJ, Yan XL, Jia YL, Wang JX, Zhao AH, Li ZW (2015). MicroRNA-125b attenuates epithelial-mesenchymal transitions and targets stem-like liver cancer cells through small mothers against decapentaplegic 2 and 4. Hepatology.

[34] Emmrich S, Rasche M, Schöning J, Reimer C, Keihani S, Maroz A, Xie Y, Li Z, Schambach A, Reinhardt D, Klusmann JH (2014). miR-99a/100~125b tricistrons regulate hematopoietic stem and progenitor cell homeostasis by shifting the balance between TGFβ and Wnt signaling. Genes Dev.

